# Access to preventive health assessments for people with intellectual disability: a systematic scoping review informed by the Levesque Access Framework

**DOI:** 10.1186/s12913-025-13060-6

**Published:** 2025-07-01

**Authors:** Maddison Eveleigh, Jodie Bailie, Alison Laycock, Sally Hall Dykgraaf, Paul Caltabiano, Bradley Shea, Nicholas Lennox, Kanchana Ekanayake, Ross Stewart Bailie

**Affiliations:** 1 Faculty of Medicine and Health, The University of Sydney, Camperdown, Australia; 2 University Centre for Rural Health, The University of Sydney, 61 Uralba Street, Lismore, Australia; 3 Centre for Disability Research and Policy, The University of Sydney, Camperdown, Australia; 4 Rural Clinical School, The Australian National University, Canberra, ACT Australia; 5 School of Rural Health, The University of Sydney, Orange, Australia; 6 Lismore Base Hospital, Lismore, Australia; 7 Queensland Centre for Intellectual and Developmental Disability, Mater Research Institute, Brisbane, Australia; 8 University of Sydney Library, The University of Sydney, Camperdown, Australia; 9 School of Public Health, The University of Sydney, Camperdown, Australia

**Keywords:** Preventive care, Intellectual disability, Health assessments, Primary care

## Abstract

**Introduction:**

Access to preventive care is essential for addressing the stark health inequities between people with intellectual disability and the general population. Despite evidence that structured annual preventive health assessments in primary care can support the delivery of evidence-based care to people with intellectual disability, their uptake remains low. This scoping review synthesises the literature examining the multiple dimensions of access to health assessments to identify the supply- and demand-side factors that are impacting on people with intellectual disability’s access to them.

**Methods:**

This scoping review followed JBI methodology. We systematically searched six databases on 15 February 2024 for relevant peer-reviewed empirical research. Two independent reviewers completed full-text screening and extracted data, mapping it to Levesque and colleagues’ theoretical framework of access, which incorporates supply-side features of health systems and services, and demand-side characteristics of consumers and populations.

**Results:**

Of the 1639 identified publications, 40 met the inclusion criteria, all originating from six high-income countries. Most of the published research focused on supply-side factors, such as the availability and promotion of assessments by health services, practitioners’ views on their effectiveness; communication challenges; and the provision of reasonable adjustments. Less frequently included were demand-side factors such as communication challenges between the practitioners and the person with intellectual disability, the health literacy both of people with intellectual disability and their carers/supporters, and the level of involvement in, and engagement with, the process by carers/supporters.

**Conclusion:**

This review systematically assesses the supply and demand-side factors impacting on access to health assessments for people with intellectual disability. Supply-side factors are well-documented, and findings on these factors show some consistency across multiple reviews, including this one. There is a need for further research to explore the perspective of practitioners who do not currently offer health assessments. Demand-side factors remain less explored than supply-side factors and warrant further investigation and research is needed to understand the perspectives of people with disability who do not currently access health assessments.

**Supplementary Information:**

The online version contains supplementary material available at 10.1186/s12913-025-13060-6.

## Introduction

People with intellectual disability experience higher rates of morbidity [1], mortality [2, 3] and potentially preventable hospitalisations compared with the general population [4]. With an estimated 240 million people with intellectual disability worldwide, these disparities represent a significant global health concern [5, 6]. People with intellectual disability typically need more support in navigating the health system compared to other groups [7]. Ensuring access to preventive health care is widely recognised as key to improving health outcomes for this population [8].

Although preventive health care may be addressed during any primary care visit, structured annual preventive health assessments (herein referred to as ‘health assessments’) are becoming more widely used to improve the delivery of evidence-based preventive care. Health assessments provide a comprehensive health evaluation of medical history, physical examination of all systems, and ensure screening for vision, hearing, and cancer are current. They also enable the identification of risk factors for chronic diseases to facilitate preventive care and early intervention [9, 10].

For several decades, health assessments targeting people with intellectual disability have been a feature of health policy in several high-income countries, such as Australia [11, 12] and the United Kingdom (UK) [13]. Health assessments for people with intellectual disability have been used to identify unrecognised health conditions [14–17], to improve the management of existing conditions [18, 19], and to facilitate health promotion [15–17, 19, 20]. For example, a study in Wales examining the medical records of 26 954 people found that health assessments were associated with reduced mortality both for people with Down Syndrome and for those with autism co-occurring with intellectual disability [21]. Despite this emerging evidence as to their effectiveness of these assessments, and efforts to promote them assessments at the primary care level, their uptake remains low [22, 23].

Three reviews have highlighted the barriers to uptake of these assessments, and all primarily focus on dimensions of healthcare access [24–26]. Barriers include lack of awareness among general practitioners and practice nurses (herein referred to collectively as practitioners) of health assessments and their benefits; limited training and experience by practitioners in the delivery of health assessments; communication challenges; and insufficient time for practitioners to complete assessments.

**Fig. 1:**
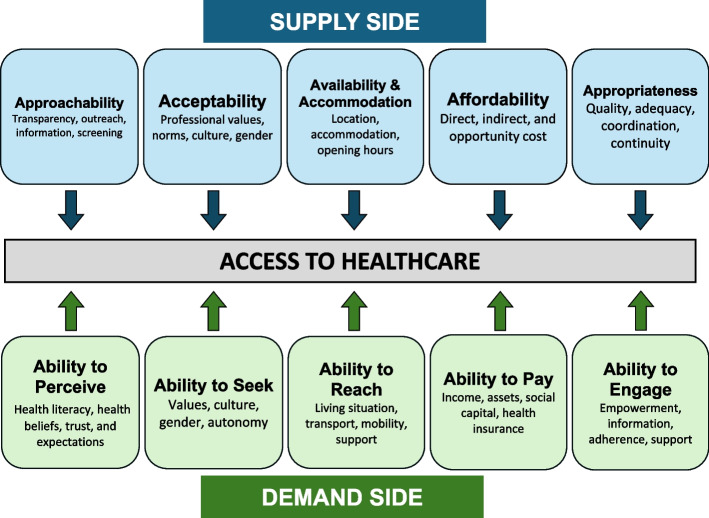
Conceptual framework of access to health care, adapted from Levesque and colleagues [32]

Over recent decades, various frameworks have been developed to understand healthcare access [27–31]. The Conceptual Framework of Access to Healthcare (herein referred to as the ‘Levesque Access Framework’), published in 2013 by Levesque and colleagues’ [32], conceptualises access as being achieved through an interaction of factors on the ‘supply-side’ (health services) and ‘demand-side’ (health care users) (Fig. 1) [32]. Supply-side factors are defined as: approachability, acceptability, availability and accommodation, affordability, and appropriateness. The corresponding demand-side factors follow a patient’s journey through accessing health care and are defined as: ability to seek, ability to perceive, ability to reach, ability to pay, and ability to engage. The Levesque Access Framework has previously been used to examine factors impacting healthcare access for people from socially vulnerable backgrounds [7, 33–35], including those with intellectual disability [7, 36, 37].

To date, a holistic framework that incorporates both supply- and demand-side dimensions has yet to be applied to understand the full range of factors impacting access to health assessments in primary care for people with intellectual disability. Our review addresses this evidence gap by assessing the published literature in this area using the Levesque Access Framework to identify these supply- and demand-side factors impacting on access to health assessments for people with intellectual disability in primary care. We anticipate that our review findings will contribute to a greater understanding of access barriers and facilitators, and guide further research on developing strategies to address these on both the supply and the demand side.

## Methods

A scoping review methodology was selected to comprehensively identify and map existing research on supply- and demand-side factors influencing access to health assessments. Our review drew on the methodology developed by JBI (formerly known as Johanna Briggs Institute) and was conducted according to a published a priori protocol [38]. Reporting was guided by the Preferred Reporting Items for Systematic Reviews and Meta-Analyses Extension for Scoping Reviews (PRISMA-ScR) checklist [39]. Consistent with JBI guidelines for scoping reviews, neither critical appraisal of quality nor risk of bias were assessed.

### Research question

The research question was: *“What supply and demand side factors impact access to structured annual preventive health assessments for people with intellectual disability in primary care?”.*

### Inclusion criteria

The scoping review parameters were defined using the “PCC—Population, Concept, Context” framework outlined in the JBI guidelines. The population of interest are people with intellectual disability, defined as having permanent decreased intellectual function, present during developmental periods and before age 18. All age groups were included. People with cerebral palsy, autism or other neurodevelopmental disorders are only included if they have a co-existing intellectual disability.

The concept of interest for the review was the determinants of access to structured annual preventive health assessments in primary care, which encompasses features of services and characteristics of users that may act as barriers or enablers to access. Publications relating to screening for specific conditions such as cervical screening and mammography are to be excluded.

The context was general practice, family medicine or primary care, in all countries. Empirical peer-reviewed research studies with any design were included. We excluded publication that were not peer-reviewed, including commentaries, perspectives, opinion, editorials, letters to the editor, books, book chapters, conference abstracts and proceedings, theses, and reviews (such as scoping and systematic reviews). Grey literature sources were excluded.

### Relevant literature search

An initial search of Medline and Google Scholar was conducted to identify key peer-reviewed publications on the topic and to develop a list of search terms. Subsequently, a full Medline search strategy was developed with an academic librarian (KE) and experts in the field of intellectual disability and health services research (JB, SHD, NL, RB) (Table 1). The Medline search terms were then translated to Embase, PsycINFO, CINAHL, Scopus and Web of Science. Database searches were from database inception to 15 February 2024. The final search strategy for each database can be found in Appendix 1.

**Table 1:** Medline search strategy, showing the population, context and concepts

*Population*	1 exp Intellectual Disability/or Fragile X Syndrome/or Rett Syndrome/or Angelman Syndrome/ 2 (Intellectual Disabilit* or intellectual disorder* or Cri-du-Chat Syndrome* or down syndrome* or de lange syndrome* or Mental retardation* or Rubinstein-taybi syndrome* or trisomy 13 syndrome* or WAGR syndrome* or Williams Syndrome* or Prader-Willi syndrome* or genetic disorder* or Fragile X* or Rett* or Angelman* or cat cry syndrome* or happy puppet syndrome*).mp. 3 Developmental Disabilities/ 4 developmental disabilit*.mp. 5 Learning Disabilities/ 6 learning disabilit*.mp. 7 (mental handicap* or Mental deficien* or mental retard*).mp. 8 1 or 2 or 3 or 4 or 5 or 6 or 7
*Context*	9 exp Primary Health Care/ 10 Primary* care*.mp. 11 Preventive Medicine/ 12 preventative medicine*.mp. 13 exp General Practice/ 14 (General practice* or Family practice* or GP service* or family doctor* or family medicine*).mp. 15 health services/or health services for persons with disabilities/or health services, indigenous/or preventive health services/ 16 (health service* or"health services for persons with disabilit*"or Indigenous health service* or preventive health service*).mp. 17 Community Health Services/ 18 community* health service*.mp. 19 Aboriginal health service*.mp. 20 nurse practitioners/or family nurse practitioners/ 21 (nurse practitioner* or family nurse practitioner*).mp. 22 Primary Care Nursing/ 23 primary care nurs*.mp. 24 Physicians, Family/ 25 family physician*.mp. 26 9 or 10 or 11 or 12 or 13 or 14 or 15 or 16 or 17 or 18 or 19 or 20 or 21 or 22 or 23 or 24 or 25
*Concept*	27 Health check*.mp. 28 Health assessment*.mp. 29 Preventive health check*.mp. 30 Health Screen*.mp. 31 27 or 28 or 29 or 30 32 8 and 26 and 31

### Study selection

Search results were uploaded into COVIDENCE [40], a web-based review platform, and duplicates removed for screening. Following a pilot test of the eligibility criteria, title and abstracts were screened by one reviewer (JB), with a randomly allocated sample of 20% sampled by another reviewer (AL). At full-text screening, three reviewers (ME, JB, AL) independently screened all publications using predefined inclusion and exclusion criteria. Throughout the review process, conflicts were resolved by JB or, if required, through further discussion between reviewers.

### Data extraction

A data extraction template was developed within COVIDENCE. The template was developed a priori and piloted on five randomly selected publications to ensure all relevant results were extracted. The template was updated after the pilot process. The template captured authors, year of publication, country and region, study setting and population, study design, methods, aims and factors influencing access to health assessments. Data extraction was performed independently by two reviewers (ME, JB). Where extracted data differed between the reviewers, consensus was reached through discussion.

### Data analysis and presentation

As data were extracted, ME and JB independently applied deductive coding using the Levesque Access Framework. This was achieved by ME and JB reading the whole publication and then, line by line, considering the relevance of the text to each dimension in the Levesque Access Framework, and coding it to the relevant domains. To guide this process, we developed a codebook reflecting our conceptualisation of access to health assessments, based on the Framework’s dimensions (outlined in Table 2), which was regularly updated throughout data extraction and analysis. ME and JB met regularly throughout this process and resolved any differences through discussion.

**Table 2:** Levesque and colleagues’ dimensions and how we conceptualised these in this review

	**Levesque Access Framework dimensions**	**How we conceptualised in our review**
Supply side dimensions	Approachability	Relates to whether people with intellectual disability and practitioners are aware that health assessments exist. Primary care practices may actively promote health assessments through patient identification, invitation, and recall.
	Acceptability	Relates to practitioners’ view of health assessments including their confidence and role in providing them and the judged appropriateness and importance of these assessments.
	Availability and Accommodation	Availability relates to whether practitioners have enough time to complete a health assessment, which encompasses the availability of other primary care staff i.e. nurses and administrative staff. Accommodation relates to the provision of reasonable adjustments to a health assessment environment and appointment and wait times to receive an appointment.
	Affordability	Relates to the adequacy of practitioner’s remuneration for delivering a health assessment.
	Appropriateness	Relates to the quality of the health care encounter and follow up care, including fit between the service and the person’s need, communication, including practitioner expertise and training and suitability of written information provided, and suitability of the health assessment proforma.
Demand side dimensions	Ability to perceive	Relates to the health literacy and knowledge of people with intellectual disability and their support workers/carers and therefore their recognition of the need for, and potential benefits of preventive care.
	Ability to seek	Relates to the concepts of personal autonomy and capacity to choose to seek a health assessment, knowledge about health assessments including the steps and procedure involved in undertaking an assessment, and the related medical anxiety associated with attending a primary care practice.
	Ability to reach	Relates to the capacity of people to book and attend a health assessment, including transport, personal mobility, and occupational flexibility that would enable one person to physically reach a primary care practice.
	Ability to pay	Relates to people’s and support systems’ ability to generate economic resources– through income, savings, borrowing, or loans to pay for health assessments.
	Ability to engage	Relates to the participation and involvement of a person with intellectual disability and their support worker/carer in decision-making and treatment decisions, which in turn is strongly determined by capacity and motivation to participate in care and commit to its completion.

Data coded according to the dimensions of the Levesque Access Framework were analysed through a recursive process, following the steps of content analysis as outlined by Elo and Kyngäs [41]. Specifically:

1. ME and JB independently immersed themselves in the extracted data, reading and re-reading to gain a general understanding of the content that had been deductively coded to the dimensions.
2. Within each dimension ME coded data as barriers or facilitators, writing notes and headings describing the content. ‘Barriers’ were defined as impeding access to health assessments, and ‘facilitators’ were defined as improving access to health assessments.
3. Building on the categorisation of barriers and facilitators, ME, in consultation with JB and AL, developed higher-level ‘factors’ that described each barrier and/or facilitator.
4. ME, JB, and AL refined the barriers, facilitators, and factors within each dimension by comparing, rereading, and revisiting the source publications to review the context.

During this process, ME regularly conferred with JB and AL to discuss, reflect on and cross-check the emerging factors of the analysis to ensure consistency and conceptual clarity. All authors, drawing on their experience, checked the results against their understanding of how targeted preventive health assessments were implemented in primary care, and the barriers to their implementation.

## Results

### Search results and study selection

**Fig. 2:**
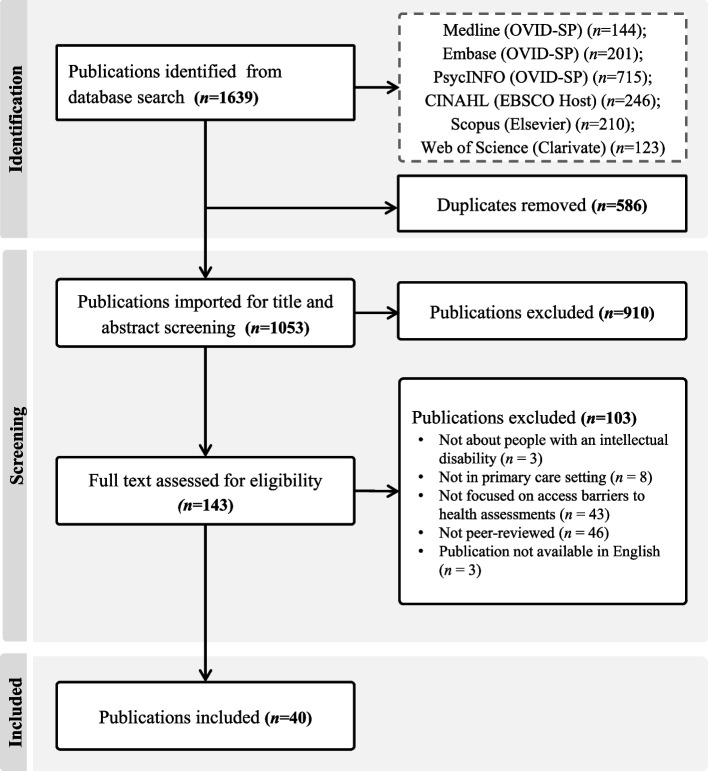
Preferred Reporting of Items for Systematic Reviews and Meta-Analysis Extension for Scoping Reviews (PRISMA-ScR) flow diagram

The search identified 1639 publications. After duplicate removal, title and abstract screening, and full-text review, 40 publications were included (Fig. 2).

### Characteristics of included studies

The characteristics of the 40 included publications are presented in Table 3. Most publications had a qualitative study design (*n* = 21), 11 had a quantitative design, six were mixed methods (with integration of qualitative and quantitative data), and two included both a qualitative and quantitative design without integration. All publications were conducted in one of six high-income countries, predominantly the UK (*n* = 27), followed by Canada (*n* = 5), the Netherlands (*n* = 3), Australia (*n* = 3), New Zealand (*n* = 1), and Norway (*n* = 1). Over half of the included publications were published in the last 10 years (*n*= 21). Nine included rural and regional perspectives, but only two of these were based in a rural or remote setting [42, 43], and none presented results disaggregated by rurality. Two publications from one study had people with intellectual disability on the research team [44, 45]. Most included the perspectives both of primary care practitioners (such as general practitioners and/or practice nurses) who had implemented health assessments, and of people with intellectual disability who had undergone a health assessment (*n* = 29).

**Table 3:** Characteristics of included publications

**First author (year)**	**Study location; rurality (if stated**	**Study design; methods; study dates (if available)**	**Study population**	**Health assessments currently being implemented in practice/s when study performed**	**Dimensions of Levesque Access Framework**^a^									
					**Supply side dimensions**					**Demand side dimensions**				
					**1**	**2**	**3**	**4**	**5**	**6**	**7**	**8**	**9**	**10**
Anderson K and Jones J. (2015) [46]	South England, UK	Mixed-methods; Audit of completed health checks and questionnaire; Apr 2014– Mar 2015	33 practices; 109 people with ID, carers/supporters, supports, advocacy staff.	Yes	◎	◎	◎		◎					●
Bakker-van Gijssel E, et al. (2017) [47]	The Netherlands; 26% rural, 57% urban, 17% other	Qualitative; Focus group; Dec 2014– Feb 2015	23 GPs	No	●	◎	◎	◎	◎					●
Bakker-van Gijssel E, et al. (2018) [48]	The Netherlands	Qualitative & Quantitative; Delphi consensus study with sequential online questionnaire	20 GPs, 18–20 ID physicians	Not stated	●		〇		〇					
Bakker-van Gijssel E, et al. (2020) [49]	The Netherlands	Qualitative; Interview	14 people with ID, with 9 accompanied by a carer/supporters	Yes					●					●
Bollard M. (1999) [50]	UK	Mixed-methods; Semi-structured interview, questionnaire, audit of completed health assessments; 1997–1998	12 practices (13 GPs and PNs)	Interview before and after implementation		◎	〇		◎					●
Bond L, et al. (1997) [51]	Gwent, Wales and Gloucestershire, England, UK	Qualitative; Questionnaire; 1994—1995	125 Welsh, 132 English GPs	No		◎								〇
Burton H and Walters L. (2013) [42]	Adelaide and Riverland region, Australia; 100% Rural	Qualitative; In-depth interview	4 GPs, 8 people with ID, 6 carers/supporters	Yes	◎	◎	◎	〇	◎	〇	●	●		〇
Buszewicz M, et al. (2014) [52]	England, UK	Quantitative; Longitudinal cohort study using data from regional primary care database; 2009—2011	270 practices (9610 people with ID)	Yes	〇			〇					●	
Cassidy G, et al. (2002) [53]	‘Midlands market town’, England, UK	Mixed methods; Audit of completed health assessments, interview, questionnaire	17 people with ID, 23 carers/supporters	Yes	〇		●	●	●		●		●	
Cavanagh D, et al. (2024) [54]	Wales, UK; Rural and urban	Qualitative; Longitudinal, in depth, semi structured interviews	12 people with ID	Yes	●	◎			◎	◎		●		◎
Chambers R, et al. (1998) [55]	North Staffordshire, England, UK	Quantitative; workshops, audit of GP contact records; Nov 1995– Dec 1996	18 practices, (115 people with ID)	No	●		●							
Chapman J. (2012) [56]	Portsmouth, England, UK	Qualitative; Workshops	GPs (number not stated)	GPs from both types of practices	〇	●			◎	●				
Chinn D. (2020) [57]	London, England, UK	Qualitative; Observation and conversation analysis, reflective interview; Jul 2016– Jul 2017	8 practices (32 people with ID)	Yes		●			●					
Chinn D, (2021) [58]	London, England, UK	Qualitative; Observation and conversation analysis; Jul 2016– Jul 2017	10 practices (9 GPs, 4 PNs)	Yes					〇					◎
Chinn D. (2022) [59]	England, UK	Qualitative; Observation and conversation analysis	6 GPs, 3 PNs, 24 people with ID, 17 carers/supporters	Yes					〇					〇
Codling M and Solomon J. (2007) [60]	Wokingham area of Berkshire West Primary Care Trust, England, UK	Quantitative; Audit of completed health assessments	12 practices (70 people with ID health assessments)	Yes	●		●		●			〇		●
Durbin J, et al. (2016) [61]	Ontario, Canada	Qualitative; Focus group; implementation log; review of intervention template	2 practices (4 GPs, 1 admin staff, 1 quality manager, 2 facilitators)	No	◎				〇					
Durbin J, et al. (2019) [20]	Ontario, Canada	Mixed methods; Retrospective chart review and questionnaire; Jul 2013– Aug 2015	139 people with ID health assessments, 147 practice staff	Yes	◎	◎	●	●	〇					
Fredheim T, et al. (2013) [62]	Hedmark Country, Norway; 60% Rural and 40% urban	Qualitative; interviews; Oct– Nov 2011	10 GPs	No		●								
Glover G, et al. (2013) [63]	England, UK	Quantitative; Analysis of data from English Primary Care Trusts; 2008—2012	All practices in region	Yes	●									
Hatton C, et al. (2024) [64]	UK	Quantitative; Longitudinal cohort study; Structured online interview, online survey; 2020–2022 (COVID-19 pandemic)	550 people with ID and carers/supporters as proxies	Yes			〇							
Kerr M, et al. (1996) [65]	Gwent, South Wales, UK	Quantitative; Postal questionnaire; 1994	126 GPs	No		◎								
Lennox N, et al. (2001) [66]	Queensland, Australia.	Mixed methods; Case note audit, self-evaluation	45 GPs participated (15 completed all components of study)	No		〇			〇					
Lennox N, et al. (2013) [67]	Greater Brisbane, Queensland, Australia	Qualitative; Telephone interviews; Aug 1998– Sep 2000	46 GPs	Yes		◎	●		〇			●		●
Lodge K, et al. (2011) [68]	UK	Quantitative; Audit of electronic medical records	1 practice (64 people with ID)	Yes	●									
MacDonald S, et al. (2018) [69]	Greater Glasgow and Clyde, Scotland, UK	Qualitative; Semi-structured interview; Mar– April 2012	11 PNs	No		◎	●		●					
Martin D, et al. (1997) [70]	UK	Mixed methods; Audit of case notes, forum, questionnaire, focus group	52 people with ID, 104 carers/supporters, 36 ‘service users’	Yes		◎	●		◎					
McConkey R, et al. (2002) [71]	Down Lisburn HSS Trust, UK; 35% rural, 65% urban	Qualitative & Quantitative; Postal questionnaire before and after intervention	Pre: 74 GPs Post: 91 GPs	No	◎	◎	〇		◎					
McConkey R, et al. (2015) [72]	Northern Ireland, UK	Quantitative; Audit of claims for health assessment data; 2011—2014	351 practices (1944 people with ID)	Yes	◎	●		●				〇		
McNeil K, et al. (2023) [73]	Nova Scotia, Canada	Qualitative; Semi-structured online interview, Online focus group; May 2021– September 2022	10 people with ID (4 supported by carers) 9 GPs, 2 Nurse practitioners, 8 family members, 5 administration staff	Yes	◎	〇	◎	●	●	◎	●	●		●
Michell B (2011) [45]	Oxfordshire; UK	Qualitative; Interview; Inclusive research	5 practices	Yes	●	◎	●		●					●
Perry J, et al. (2014) [74]	Wales, UK; Rural and urban	Qualitative; Focus group	39 people with ID	Yes	◎	〇	●			◎				
Ratanyake I, et al. (2021) [75]	Manitoba, Canada; Rural and urban	Quantitative; Retrospective cohort study using linked administrative health and non-health data; 2009/10 and 2014/15	9567 people with ID	Yes	〇	●					〇	●		
Romeo R, et al. (2009) [76]	Greater Glasgow Health Board Area; Scotland, UK	Quantitative; Economic analysis with matched control group	100 people with ID	Yes				〇						
Russell A, et al. (2017) [77]	West Yorkshire, England, UK	Quantitative; Audit of electronic medical record	145 practices (325 people with ID identified)	n/a	●									
Shooshtari S, et al. (2017) [78]	Manitoba, Canada	Qualitative; Semi-structured interview, focus group	Interviews- 2 GPs, 3 PNs, 13 carers/supporters; Focus groups– 7 GPs, 11 team leaders/carers	Yes		◎	●	●	◎					
Taylor H, et al. (2023) [79]	Northeast England, UK	Qualitative; Focus group, interview; 2018—2020	8 people with ID, 6 people with co-occurring ID and autism, 5 ‘primary care professionals’, 7 carers/supporters	Yes	◎	●	〇	●	●					〇
Walmsley J (2011) [44]	Oxfordshire England, UK; 33% rural, 66% urban	Qualitative; Interview, questionnaire; Inclusive research; 2010	6 practices	Yes	◎	◎	●	●	●					
Webb O and Rogers L (1999) [80]	New Zealand	Qualitative; Document review and audit of health assessments	1311 people with ID, GPs and health care staff (number not stated)	Yes	●	◎	◎	●	〇	●			〇	
Wigham S, et al. (2022) [43]	UK; 100% Regional	Qualitative; Online focus group, online interview, open response survey; Jun– Dec 2021	16 participants (8 GPs or PNs, 4 people with ID, 4 family members)	Yes	◎	〇	◎		◎	●	●			◎
Total number of publications					25	26	22	12	27	7	5	7	3	15

*GP* General Practitioner, *PN* Practice Nurse, *ID* Intellectual Disability, *UK* United Kingdom ● = barrier. 〇 = facilitator. ◎ = barrier and facilitator

^a^Levesque Access Framework supply side access dimensions: 1– approachability, 2– acceptability, 3– availability and accommodation, 4– affordability, 5 appropriateness. Demand side access dimensions 6– ability to perceive, 7– ability to seek, 8– ability to reach, 9– ability to pay, 10– ability to engage

### Barriers and facilitators to accessing preventive health assessments

Nineteen publications identified factors solely impacting the supply-side dimensions of access (characteristics of health services), seven focused solely on factors impacting demand-side dimensions (characteristics of people with intellectual disability), and 14 identified factors related both to supply- and demand-side dimensions.

### ‘Approachability’ and ‘ability to perceive’

Twenty-five publications identified supply-side factors that impacted on the approachability of health assessments which, in the context of this review, mainly encompasses the promotion of health assessments (Table 4). Primary care practices often failed to provide or promote health assessments [20, 42–44, 46, 47, 52–56, 61, 68, 71–73, 75], in part due to the inability of their clinical systems to identify patients with intellectual disability, thereby limiting their ability to identify eligible patients and invite or recall them for a health assessment [43, 44, 46, 47, 61, 63, 68, 73, 77, 79]. Some primary care practices promoted health assessments through routine invitations and reminders to attend [20, 44, 46, 52, 53, 56, 61, 72–75], but these were not always issued in ways that were clear and accessible to people with intellectual disability [44, 45, 54, 60, 74, 80], Further barriers included practitioners having little or no knowledge of health assessments and/or of the associated preventive health guidelines [20, 42, 73].

**Table 4:** Factors impacting access to health assessments for people with intellectual disability, paired dimensions of ‘approachability’ and ‘ability to perceive’

Levesque Access Framework dimensions	Factors	Barriers	Facilitators
Approachability [20, 42–48, 52–56, 60, 61, 63, 68, 71–75, 77, 79, 80] (*n* = 25)	Provision and promotion of health assessments [20, 42–44, 46, 47, 52–56, 61, 68, 71–73, 75] (*n* = 17)	Primary care not providing [42, 55, 71] or not promoting health assessments to people with ID [43, 44, 46, 47, 54, 55, 68, 72, 73] (*n* = 11)	Primary care actively promoting health assessments to people with ID [20, 44, 46, 52, 53, 56, 61, 72–75] (*n* = 12)
	Accessibility of information about health assessment provided to people with ID [44, 45, 54, 60, 73, 74, 79, 80] (*n* = 8)	Primary care providing little information on health assessments [44, 45, 60, 74, 80] or not providing this information in an accessible format [44, 45, 54] (*n* = 6)	Accessible information on health assessment available at primary care [43, 61, 73, 79] (*n* = 4)
	Capacity of primary care clinical information systems to identify people with ID [43, 44, 46, 47, 52, 61, 63, 68, 73, 77, 79] (*n* = 11)	Challenges to, or failure of, clinical information systems to identify people with ID [43, 44, 46, 47, 61, 63, 68, 73, 77, 79] (*n* = 10)	Clinical information systems capable of identifying people with ID [52] (*n* = 1) List of relevant ID syndromes and conditions developed to improve identification of ID [46] (*n* = 1)
	Degree to which health assessment proforma is electronically compatible with primary care existing software [48, 73] (*n* = 2)	Incompatibility of health assessment proforma with practices’ existing electronic software and tools [48, 73] (*n* = 2)	
	Level of practitioners’ awareness of health assessments and guidelines for preventive care [20, 42, 45, 46, 71, 73] (*n* = 4)	Practitioners have limited or no knowledge of health assessments and/or preventive care guidelines [20, 42, 73] (*n* = 3)	Practitioners are familiar with health assessments and preventive care guidelines [42, 71] (*n* = 2)
Ability to perceive (*n* = 7)	Health literacy levels of people with ID and their carers/supporters [42, 43, 54, 56, 73, 74, 80] (*n* = 7)	People with ID and/or carers/supporters do not understand the importance or benefits of health assessments [43, 54, 56, 73, 74, 80] (*n* = 6)	People with ID and carers/supporters do understand the importance and benefits of health assessments [42, 54, 73, 74] (*n* = 4)

*ID* Intellectual disability

Seven publications identified demand-side factors impacting on the ability to perceive, which was conceptualised by whether people with intellectual disability and their carers/supporters value health assessments. Mixed views were identified, with some individuals perceiving limited value or significance in health assessments [43, 54, 56, 73, 74, 80], while others recognised them as a useful approach for maintaining good health [42, 54, 73, 74].

### ‘Acceptability’ and ‘ability to seek’

Twenty-six publications identified supply-side factors impacting the acceptability of health assessments, a dimension that encompassed practitioner perspectives and continuity of care (Table 5). There were several reasons that practitioners viewed health assessments negatively: there is a lack of evidence they improve long-term health outcomes [42, 44, 45, 47, 56, 62, 67, 69, 71, 72, 75, 80]; they are seen as too time intensive [50, 67, 69–72, 78, 80]; and there is uncertainty around the practitioners role in providing them [51, 65, 67, 69–71, 78, 79]. Some also felt unprepared or lacked confidence in carrying out a thorough health assessment for people with intellectual disability [20, 42, 44, 47, 50, 57, 69]. Conversely, some practitioners did see health assessments as a positive intervention [20, 42, 44, 47, 50, 66, 67, 69, 78]. Another factor identified in this dimension was the degree of continuity of care between practitioners and people with intellectual disability. A long-term professional relationship, in which the practitioner is familiar with the person, facilitated access to health assessments [42, 45, 46, 54, 69, 70, 73, 74].

**Table 5:** Factors impacting access to health assessments for people with intellectual disability, paired dimensions of ‘acceptability’ and ‘ability to seek’

Levesque Access Framework dimensions	Factors	Barriers	Facilitators
Acceptability [20, 42–47, 50, 51, 54, 56, 57, 62, 65–67, 69–75, 78–80] (*n* = 26)	Degree of continuity of care between practitioners and people with ID [42, 45, 46, 54, 69, 70, 73, 74] (*n* = 8)	Practitioners being unfamiliar to person with ID lessens the likelihood of them completing the assessments [54] (*n* = 1)	Continuity of care between practitioners and people with ID increases the likelihood of receiving a health assessment [42, 45, 46, 54, 69, 70, 73, 74] (*n* = 8)
	Practitioners’ comprehension of health burden experienced by people with ID [20, 44, 47, 65–67] (*n* = 6)	Practitioners unaware of the increased health burdens faced by people with ID [20, 44, 47] (*n* = 3)	Practitioners are informed about the increased health burden faced by people with ID [20, 65–67] (*n* = 4)
	Practitioners attitude to, and perception of, health assessments [20, 42, 44, 45, 47, 50, 56, 62, 66, 67, 69–72, 75, 78, 80] (*n* = 17)	Practitioners’ views on efficacy and benefits of health assessments are unfavourable [42, 44, 45, 47, 56, 62, 67, 69, 71, 72, 75, 80] (*n* = 12) Practitioners believe that health assessments are too time intensive to be implemented [50, 67, 69–72, 78, 80] (*n* = 8)	Practitioners view health assessments as a favourable, effective clinical tool in which to invest time and resources [20, 42, 44, 47, 50, 66, 67, 69, 78] (*n* = 9)
	Practitioners level of confidence in their ability to conduct health assessments [20, 42, 44, 46, 47, 50, 57, 69, 78, 80] (*n* = 10)	Practitioners feeling unprepared, incompetent and/or have low confidence in either their own or other practitioners’ abilities to conduct health assessments [20, 42, 44, 47, 50, 57, 69] (*n* = 7)	Adequate confidence in both their own and other practitioners’ ability to conduct health assessments [20, 42, 69, 78] (*n* = 4) Increased use of health assessments by practitioners improves their confidence in providing care to people with ID [20, 46, 67, 78, 80] (*n* = 5)
	Practitioners’ views regarding their role in providing health assessments [42, 43, 47, 50, 51, 65, 67, 69–71, 78, 79] (*n* = 13)	Practitioners unsure about [67, 78] or disagree with their role/responsibility in providing health assessments to people with ID in primary care [51, 65, 69–71, 78, 79] (*n* = 9)	Practitioners agree that they have a role and responsibility to provide health assessments to people with ID in primary care [42, 43, 47, 50, 51, 71, 78] (*n* = 7)
Ability to seek [42, 43, 53, 73, 75] (*n* = 5)	Level of medical anxiety for people with ID regarding processes or outcomes of the health assessment [42, 43, 53, 73] (*n* = 4)	Person with ID avoiding a health assessment due to anxiety towards discomfort during health assessment, [53] a fear of doctors or health professionals in general, [42, 43] or worry about receiving news about being ill [42, 73] (*n* = 4)	
	People with ID and carers/supporters level of knowledge and experience of health assessments and their availability [42, 43, 75] (*n* = 3)	People with ID and/or carers/supporters unaware that health assessments are available [42, 43] (*n* = 2)	A history of regular successful health assessments [75] (*n* = 1)

*ID* Intellectual disability

Five publications identified factors impacting on the demand-side dimension of ability to seek, which encompassed people’s knowledge of health assessments. Some people with intellectual disability did not wish to seek a health assessment due to medical anxiety, including the worry of discovering they are ill [42, 73], anxiety regarding potential discomfort such as injections [53], or a more general fear of doctors and/or health professionals [42, 43]. Others were simply unaware of the existence of health assessments [42, 43], thereby limiting their access to them. One publication reported a previous health assessment to be a positive predictor of whether someone accessed a future assessment [75].

### ‘Availability and accommodation’ and ‘ability to reach’

Twenty-two publications identified supply-side factors impacting the availability and accommodation of health assessments (Table 6). In the context of this review, availability and accommodation were conceptualised by the provision of reasonable adjustments and practitioner time to complete a health assessment. Practitioners reported that health assessments take longer to prepare for and conduct than a standard consultation [20, 42, 46, 47, 55, 67, 69, 70, 78, 80]. Some primary care practices had employed a practice nurse to assist with health assessments which was effective in relieving time pressure on general practitioners [42, 47, 48, 73]. Situations that can discourage a person from undertaking a health assessment include long wait times in the waiting room [46, 53, 70, 73, 74], as these can be anxiety-provoking due to sensory needs [43, 46, 73], and/or clinics having inaccessible spaces for people with mobility needs [60, 73]. Some primary care practices and practitioners were unaware of what constitutes a reasonable adjustment for people with intellectual disability [43–45], but others did see the value in offering reasonable adjustments to health assessments [50, 71], such as home visits [44, 46] and flexible appointment times [43, 46, 64, 79, 80].

**Table 6:** Factors impacting access to health assessments for people with intellectual disability, paired dimensions of ‘availability and accommodation’ and ‘ability to reach’

Levesque Access Framework dimensions	Factors	Barriers	Facilitators
Availability and accommodation [20, 42–48, 50, 53, 55, 60, 64, 67, 69–71, 73, 74, 78–80] (*n* = 22)	Degree to which reasonable adjustments surrounding health assessment appointments are made [43–46, 50, 53, 60, 64, 70, 71, 73, 74] (*n* = 14)	Lack of awareness as to what constitutes a reasonable adjustment [43, 44], such as long wait times in the waiting room [46, 53, 70, 73, 74], overstimulating environments [43, 46, 73] or inaccessible spaces for people with mobility needs [60, 73] (*n* = 13)	Reasonable adjustments to health assessment appointments provided, [50, 71] such as flexible appointment times [43, 46, 64, 79, 80] (*n* = 7)
	Time available to prepare for and complete health assessment [20, 42, 46–48, 55, 67, 69, 70, 73, 78, 80] (*n* = 12)	Time constraints limiting practitioners’ ability to prepare for and complete health assessment [20, 42, 46, 47, 55, 67, 69, 70, 78, 80] (*n* = 11)	Employing a practice nurse to assist with health assessments to increase the capacity of the primary care practice to offer health assessments [42, 47, 48, 73] (*n* = 4)
	Length of wait time to receive a health assessment appointment [42, 46] (*n* = 2)	People with ID deterred by long wait times between booking and receiving health assessments [42, 46] (*n* = 2)	
Ability to reach [42, 53, 54, 60, 67, 72, 73] (*n* = 7)	Availability and accessibility of transport to primary care practices [42, 54, 67, 73] (*n* = 4)	Coordination of transport and journey for people with ID to access health assessments [42, 54, 67, 73] (*n* = 4)	
	Time available for person with ID to attend a health assessment amongst daily commitments such as work or school [53, 73] (*n* = 2)	People with ID concerned about missing daily activities e.g. school/work [53, 73] (*n* = 2)	
	Whether carers/supporters provide support to make a health assessment appointment [60, 72] (*n* = 2)		People with ID have the support they need– e.g. access to a carer/supporter or living in supported care– to make health assessment appointment [60, 72] (*n* = 2)

Seven publications identified factors impacting on the demand-side dimension of ability to reach, such as access to transport and the availability of a person with intellectual disability to attend a health assessment. People with intellectual disability can experience arduous coordination of their journey to an appointment [42, 54, 67, 73], particularly pertinent for those living in rural locations [42]. Other people with intellectual disability and carers/supporters expressed concerns as to how a health assessments could be accommodated within a busy schedule [53, 73]. People were also more likely to schedule a health assessment appointment if they had support [60, 72].

### ‘Affordability’ and ‘ability to pay’

Eleven publications identified a supply-side factor impacting on the affordability of health assessments, which in the context of this review was conceptualised by the adequacy of financial remuneration for primary care practitioners (Table 7). A majority of publications found that remuneration for health assessments did not reflect the time invested by practitioners in preparing and conducting the assessments [20, 44, 47, 55, 72, 73, 78–80]. Only three publications identified a perception from practitioners that health assessments were being adequately remunerated [42, 47, 52].

**Table 7:** Factors impacting access to health assessments for people with intellectual disability, paired dimensions of ‘affordability’ and ‘ability to pay’

Levesque Access Framework dimensions	Factors	Barriers	Facilitators
Affordability [20, 42, 44, 47, 52, 72, 73, 76, 78–80] (*n* = 11)	Adequacy of financial remuneration [20, 42, 44, 47, 52, 72, 73, 76, 78–80] (*n* = 11)	Practitioners believe they are inadequately remunerated to prepare for and complete health assessments [20, 44, 47, 72, 73, 78–80] (*n* = 8)	Remuneration is adequate for practitioners to prepare for and complete health assessments [42, 47, 52] (*n* = 3) The cost effectiveness of health assessments is proven [76] (*n* = 1)
Ability to pay [52, 75, 80] (*n* = 3)	Ability of person with ID or support systems to pay for health assessment [52, 75, 80] (*n* = 3)	Younger people and/or those from lower socio-economic backgrounds are less likely to undertake health assessments [52, 75] (*n* = 2)	Government subsidises cost of health assessment appointment where needed [80] (*n* = 1)

*ID* Intellectual disability

The demand-side dimension of ability to pay, which encompasses a person’s ability to pay for and afford a health assessment, was identified in three publications. Being of younger age and from a lower socioeconomic background were the greatest barriers to a person’s ability to pay [52, 75]. A facilitator for this dimension was the availability of a government subsidy to reduce the cost of the health assessment for the person undergoing a health assessment [80].

### ‘Appropriateness’ and ‘ability to engage’

Twenty-seven publications identified supply-side factors that impacted the appropriateness of health assessments, which was conceptualised by the quality of care provided to people with intellectual disability (Table 8). Additional training in health assessments appears to be important for practitioners to provide high-quality, thorough health assessments to people with intellectual disability [42, 45, 47, 50, 69–71, 73, 78]. The communication skills of practitioners were frequently reported as being insufficient or needing to be improved [42, 43, 53, 54, 73, 79] if practitioners were to interact effectively with people with intellectual disability. A similar barrier to communication was the failure to offer, or the underutilisation of, accessible health information such as Easy Read format [43–46, 49, 54, 57, 73]. Three important aspects of the effectiveness of health assessments are the action plan, referrals to further services, and follow-up. However, some publications found practitioners were not developing action plans to follow-up on identified health needs [60]. Additionally, practitioners’ knowledge of disability support services was limited [20, 42, 54, 70], with many unclear as to whose responsibility it was to provide follow-up care [54, 78], thereby reducing the quality of care given to those completing health assessments. The facilitators of appropriateness were twofold: firstly, that health assessments improved practitioner awareness and knowledge of people with intellectual disability [20, 47, 50, 67, 78, 80] so they were able to provide better care [20, 66, 78]; and secondly, that some practitioners were comfortable using patient-friendly language [42, 46, 54, 58, 59, 70].

**Table 8:** Factors impacting access to health assessments for people with intellectual disability, paired dimensions of ‘appropriateness’ and ‘ability to engage’

Levesque Access Framework dimensions	Factors	Barriers	Facilitators
Appropriateness [20, 42–50, 53, 54, 56–61, 66, 67, 69–71, 73, 78–80] (*n* = 27)	Level of practitioner’s education and experience in providing health assessments to people with ID [20, 45, 47, 50, 61, 67, 69–71, 73, 78] (*n* = 12)	Inadequate training for practitioners to provide a high-quality health assessment for people with ID [45, 47, 50, 69–71, 73, 78] (*n* = 8)	Practitioners acknowledge that health assessments improve their awareness and knowledge of how to care for people with ID and are interested in participating [20, 47, 50, 67, 78, 80] (*n* = 6) Training and support provided to practitioners who want to participate [50, 56, 61, 71] (*n* = 4)
	Suitability of the health assessment proforma and process for providing a high-quality health assessment [20, 43–45, 54, 60, 66, 67, 78] (*n* = 9)	Unsuitable or lack of health assessment proforma reduces likelihood of practitioners detecting health concerns [44, 45, 54] (*n* = 3) Incomplete health assessments miss vital health information [54, 60] (*n* = 2)	Evidence-based health assessment proforma employed which increases quality of care [20, 43, 66, 67, 78] (*n* = 5)
	Degree of effective communication established between practitioners and people with ID [42–46, 53, 54, 56, 56–59] (*n* = 15)	Communication skills of practitioners are insufficient or needing improvement [42, 43, 53, 54, 73, 79] (*n* = 6) Failure to offer or to utilise health information in an accessible format [43–46, 49, 54] (*n* = 8)	Practitioners use patient-friendly language to communicate effectively with person with ID [42, 46, 54, 58, 59, 70] (*n* = 6 )Health information provided in accessible formats [56] (*n* = 1)
	Appropriateness of care plans and actions based on health assessment findings [20, 42, 48, 50, 54, 60, 70] (*n* = 8)	No health action plan formed [60] or little health information given [54] (*n* = 2) Practitioners’ knowledge of disability services limited [20, 42, 54, 70] (*n* = 4)	Final health action plan formed [48] (*n* = 1 Practice nurse providing more appropriate referrals based on health assessment [50] (*n* = 1)
	Level of responsibility taken by practitioners for follow-up [43, 54, 78] (*n* = 3)	Concerns regarding allocation of responsibility for follow-up on required actions from health assessment [54, 78] (*n* = 2)	Practitioners acknowledge the importance of planning follow-up to the health assessment [43] (*n* = 1)
	Quality of medical records kept by primary care practice [56, 60] (*n* = 2)	Lack of clinical information on person with ID [56, 60] (*n* = 2)	
Ability to engage [42, 43, 45–47, 49–51, 54, 58–60, 67, 73, 79] (*n* = 15)	Communication challenges between person with ID and practitioner [42, 43, 46, 49–51, 54, 58, 59, 73, 79] (*n* = 11)	People with ID facing challenges to communication [43, 49, 50, 58, 73] such as difficulty recalling health issues [49] and rushed health assessments [46, 54, 73] (*n* = 7)	Effective communication achieved with support from carer/supporter if required [42, 43, 54, 58, 59, 79] (*n* = 6) People with ID able to answer practitioner’s questions [58] and is prepared for health assessement [51] (*n* = 2)
	Level of involvement and engagement of carer/supporter with person with ID [42, 45, 47, 60, 67, 73] (*n* = 6)	Carer/supporter may not recognise the importance of symptoms [47, 60] (*n* = 2) Lack of carer/supporter continuity reducing their ability to engage with and understand people with ID [45, 47, 67] (*n* = 3) Carer/supporter worrying about being silenced or looked down upon by practitioner [73] (*n* = 1)	Carer/supporter makes valuable contributions to health assessment including providing practitioner with specific communication strategies [42] (*n* = 1)

*ID* Intellectual disability

The demand-side dimension of ability to engage, which was identified in 15 publications, was conceptualised as a person with intellectual disability being able to participate with the health assessment and the practitioner. The main barriers were a lack of inclusive communication [43, 49, 73] and of continuity of carers/supporters [45, 47, 67]. The main facilitator of a person’s ability to engage is having effective communication channels to relay health information to the practitioner, supported by a carer/supporter [42, 43, 50, 54, 58, 59, 79].

## Discussion

This scoping review adds to the existing evidence base through systematically identifying supply- and demand-side factors that impact access to understand utilisation of structured annual preventive health assessments in primary care for people with intellectual disability. In our review, the most common supply-side factors were the provision and promotion of health assessments, practitioners’ views and attitudes towards the effectiveness of these assessments, the degree of effective communication established between practitioners and people with intellectual disability, and the provision of reasonable adjustments. Less frequently studied were demand-side factors such as the ability of people with intellectual disability to communicate with a practitioner, the health literacy of people with intellectual disability and their carers/supporters, and the level of involvement and engagement of the carer/supporter.

There was limited research focusing on understanding the perspectives of people with disability who do not currently access health assessments and of practitioners who do not conduct health assessments for this population. However, our review confirmed our initial impressions that there has been relatively limited attention in the peer-reviewed literature to demand-side factors affecting access to health assessments. This is an important gap because it limits the ability of services to move to a more patient-centred approach to improving health outcomes.

The factors identified in our review align with findings from recent reviews [24–26]. For example, the work of Caltabiano and colleagues focused on supply-side factors and highlighted primary care practitioners’ behaviours influencing the implementation of health assessments for this population [24]. Sonderlund and colleagues used an ecological approach to outline barriers to implementing both health assessments and screening programs; however, their review may be limited by search terms requiring the word ‘implementation’ and use of an automated pre-screening process that might have omitted some eligible studies [25]. Gregson and colleagues examined the experiences of people with intellectual disability in accessing not only health assessments, but also broader screening programs and primary care [26]. While these reviews applied different analytical frameworks, they converge on the same core issue: the low uptake of health assessments related to access issues.

Efforts to improve access to health assessments are more likely to succeed when identified barriers and facilitators are targeted [81]. Given the consistency of barriers and facilitators on the supply-side, across multiple reviews [24–26], including this review, we propose this established knowledge base could be used to co-design targeted interventions to improve access and uptake of health assessments. Simultaneously, research is undertaken into the perspectives of currently underrepresented groups—such as individuals who do not access health assessments and practitioners who do not offer them—will be essential to continuously refine and optimise these strategies.

Over two-thirds of publications were from the UK, followed by Canada, Australia, New Zealand, and Norway. The UK and Australia have national policies to support the implementation of health assessments, including the UK Direct Enhanced Service [82] and Australia’s National Roadmap to Improve Health Outcomes for People with Intellectual Disability [11]. These policies are a recognition of and response to the need to improve healthcare for people with intellectual disability, and point to the importance of high-level policy support and resourcing for implementation to enhance the delivery of preventive care [83]. Additionally, the role of advocacy organisations is crucial in increasing awareness of the availability of preventive health assessments at the community level [12]. These programs also provide essential support in building self-advocacy skills for people with intellectual disability. For example, in Australia, the Council for Intellectual Disability has created a series of Easy-Read fact sheets to build awareness of the availability of preventive health assessments [84]. While outside the scope of this review, there may be differences in healthcare systems that influence the extent to which practitioners are willing or able to work with people with intellectual disability, and these differences may have influenced the focus and amount of research relevant to the review.

The Levesque and colleagues’ Access Framework is widely cited and provided a structured approach to identifying factors impacting access to health assessments in primary care. However, it does not explicitly include a health system domain, such as system-level policy. We recommend incorporating a health system element into the framework, potentially as an overarching component. In our review, national policies appeared to influence access to preventive health assessments. Like other scholars who applied the framework [7, 33, 35, 85], we needed to continually reflect on how we were conceptualising the dimensions in our review, and applying the data to the most relevant dimension.

### Strengths and limitations

A strength of the review was the use of a published a priori protocol, which improved its transparency and reproducibility, and the use of a well-cited framework that has been previously used effectively in the context of intellectual disability previously. A further strength was the involvement of two independent reviewers at the full-text screening and data extraction stages, ensuring rigour and reliability.

The limitations of this review include the potential for missing evidence, as grey literature and publications in languages other than English were excluded. There is also potential for selection bias in the included publications, as they were predominantly focussed on practitioners who were already performing health assessments, and only a few centred-on patient perspectives. As the comprehensiveness of our review is contingent on the scope of the included publications, and not all of these have taken an approach of investigating factors impacting access to health assessments, our insights are solely based on what has been studied and thereby provide only a partial picture. Additionally, all included publications originated from six high-income countries. We did not identify studies from other countries that met our inclusion criteria, which may limit the relevance or applicability of our findings to other countries.

## Conclusion

This scoping review synthesises research on the supply and demand factors affecting access to health assessments for people with intellectual disability, using a well-cited access framework. While supply side factors are well documented, further investigation into demand-side factors is needed to understand fully what influences access. Future research should focus on practitioners and people with intellectual disability who are not currently utilising health assessments, as their perspectives are often underrepresented. Addressing this gap will provide a fuller understanding of the barriers to access from multiple perspectives. Simultaneously, given the consistent barriers and enablers related to supply-side factors across numerous reviews, including this one, efforts should now shift towards co-designing and implementing targeted interventions that leverage this established knowledge to improve access and uptake. Implementing interventions that translate this knowledge into practical strategies for improving access and uptake of health assessments holds the potential to drive lasting improvements in preventive care for this population, while ongoing research into underrepresented perspectives will further inform and refine these strategies to ensure they are comprehensive and effective.

## Supplementary Information

Supplementary Material 1.

## Data Availability

Further details on studies included in this scoping review can be retrieved by contracting the corresponding author at jodie.bailie@sydney.edu.au.
