# Supplementary material for: Access to preventive health assessments for people with intellectual disability: a systematic scoping review informed by the Levesque Access Framework
Source: BMC Health Serv Res. 2025 Jul 1;25:867. doi: 10.1186/s12913-025-13060-6 (PMC12218937; doi:10.1186/s12913-025-13060-6)
Supplement: Supplementary file 1 — Supplementary Material 1. [file 12913_2025_13060_MOESM1_ESM.docx]

Appendices

Appendix 1: Search strategy

*Table 1.1 Summary of data base search results*

| **Database** | **Result** |
| --- | --- |
| Medline | 144 |
| Embase | 201 |
| PsycINFO | 715 |
| CINAHL | 246 |
| Scopus | 210 |
| Web of Science | 123 |
| Total (with Duplicate) | 1639 |
| Total (without Duplicate) | 1053 |

**Database 1: Medline (OVID-SP) – Coverage 1946 – Present**

1 exp Intellectual Disability/ or Fragile X Syndrome/ or Rett Syndrome/ or Angelman Syndrome/

2 (Intellectual Disabilit* or intellectual disorder* or Cri-du-Chat Syndrome* or down syndrome* or de lange syndrome* or Mental retardation* or Rubinstein-taybi syndrome* or trisomy 13 syndrome* or WAGR syndrome* or Williams Syndrome* or Prader-Willi syndrome* or genetic disorder* or Fragile X* or Rett* or Angelman* or cat cry syndrome* or happy puppet syndrome*).mp.

3 Developmental Disabilities/

4 developmental disabilit*.mp.

5 Learning Disabilities/

6 learning disabilit*.mp.

7 (mental handicap* or Mental deficien* or mental retard*).mp.

8 1 or 2 or 3 or 4 or 5 or 6 or 7

9 exp Primary Health Care/

10 Primary* care*.mp.

11 Preventive Medicine/

12 preventative medicine*.mp.

13 exp General Practice/

14 (General practice* or Family practice* or GP service* or family doctor* or family medicine*).mp.

15 health services/ or health services for persons with disabilities/ or health services, indigenous/ or preventive health services/

16 (health service* or "health services for persons with disabilit*" or Indigenous health service* or preventive health service*).mp.

17 Community Health Services/

18 community* health service*.mp.

19 Aboriginal health service*.mp.

20 nurse practitioners/ or family nurse practitioners/

21 (nurse practitioner* or family nurse practitioner*).mp.

22 Primary Care Nursing/

23 primary care nurs*.mp.

24 Physicians, Family/

25 family physician*.mp.

26 9 or 10 or 11 or 12 or 13 or 14 or 15 or 16 or 17 or 18 or 19 or 20 or 21 or 22 or 23 or 24 or 25

27 Health check*.mp.

28 Health assessment*.mp.

29 Preventive health check*.mp.

30 Health Screen*.mp.

31 27 or 28 or 29 or 30

32 8 and 26 and 31

**Database 2: Embase (OVID-SP) Coverage 1947-Present**

1 intellectual impairment/

2 fragile X syndrome/

3 Rett syndrome/

4 happy puppet syndrome/

5 cat cry syndrome/

6 Down syndrome/

7 de Lange syndrome/

8 mental deficiency/

9 Rubinstein syndrome/

10 trisomy 13/

11 WAGR syndrome/

12 Williams Beuren syndrome/

13 Prader Willi syndrome/

14 genetic disorder/

15 fragile X syndrome/

16 Rett syndrome/

17 (Intellectual Disabilit* or intellectual disorder* or Cri-du-Chat Syndrome* or down syndrome* or de lange syndrome* or Mental retardation* or Rubinstein?taybi syndrome* or trisomy 13 syndrome* or WAGR syndrome* or Williams Syndrome* or Prader?willi syndrome* or genetic disorder* or Fragile X* or Rett* or Angelman* or cat cry syndrome* or happy puppet syndrome*).mp.

18 developmental disorder/

19 (developmental disabilit* or developmental disorder*).mp.

20 learning disorder/

21 (Learning Disabilit* or learning disorder*).mp.

22 mental deficiency/

23 mental retardation malformation syndrome/

24 (mental handicap* or Mental deficien* or mental retard*).mp.

25 1 or 2 or 3 or 4 or 5 or 6 or 7 or 8 or 9 or 10 or 11 or 12 or 13 or 14 or 15 or 16 or 17 or 18 or 19 or 20 or 21 or 22 or 23 or 24

26 primary health care/

27 Primary* care*.mp.

28 preventive medicine/

29 preventative medicine*.mp.

30 general practice/

31 (General practice* or Family practice* or GP service* or family doctor* or family medicine*).mp.

32 health service/

33 indigenous health care/

34 preventive health service/

35 (health service* or "health services for persons with disabilit*" or Indigenous health service* or preventive health service*).mp.

36 community care/

37 community* health service*.mp.

38 Aboriginal health service*.mp.

39 nurse practitioner/

40 family nurse practitioner/

41 (nurse practitioner* or family nurse practitioner*).mp.

42 primary health care/

43 primary care nurs*.mp.

44 general practitioner/

45 family physician*.mp.

46 26 or 27 or 28 or 29 or 30 or 31 or 32 or 33 or 34 or 35 or 36 or 37 or 38 or 39 or 40 or 41 or 42 or 43 or 44 or 45

47 Health check*.mp.

48 Health assessment*.mp.

49 Preventive health check*.mp.

50 Health Screen*.mp.

51 47 or 48 or 49 or 50

52 25 and 46 and 51

**Database 3: APA PsycINFO (OVID-SP) Coverage 1806 – current**

1            exp intellectual development disorder/ or crying cat syndrome/ or down's syndrome/ or fragile x syndrome/ or williams syndrome/

2            Rett Syndrome/

3            (Intellectual Disabilit* or intellectual disorder* or Cri-du-Chat Syndrome* or down syndrome* or de lange syndrome* or Mental retardation* or Rubinstein?taybi syndrome* or trisomy 13 syndrome* or WAGR syndrome* or Williams Syndrome* or Prader?willi syndrome* or genetic disorder* or Fragile X* or Rett* or Angelman* or cat cry syndrome* or happy puppet syndrome*).mp.

4            Developmental Disabilities/

5            developmental disabilit*.mp.

6            learning disabilities/

7            learning disabilit*.mp.

8            Mental Disorders/

9            (mental handicap* or Mental deficien* or mental retard* or mental disorder*).mp.

10          1 or 2 or 3 or 4 or 5 or 6 or 7 or 8 or 9

11          Primary Health Care/

12          Primary* care*.mp.

13          Preventive Health Services/

14          preventative medicine*.mp.

15          general practitioners/

16          (General practice* or Family practice* or GP service* or family doctor* or family medicine*).mp.

17          health care services/      5

18          Preventive Health Services/

19          (health service* or "health services for persons with disabilit*" or Indigenous health service* or preventive health service*).mp.

20          community* health service*.mp.

21          Aboriginal health service*.mp.

22          (nurse practitioner* or family nurse practitioner*).mp.

23          primary care nurs*.mp.

24          family physicians/

25          family physician*.mp.

26          11 or 12 or 13 or 14 or 15 or 16 or 17 or 18 or 19 or 20 or 21 or 22 or 23 or 24 or 25

27          Health check*.mp.

28          Health assessment*.mp.

29          Preventive health check*.mp.

30          health screen*.mp.

31          27 or 28 or 29 or 30

32          10 and 26 and 31

**Database 4: CINAHL (EBSCO Host) – Coverage 1982 – Present**

S1 (MH "Intellectual Disability+")

S2 (MH "Fragile X Syndrome")

S3 (MH "Angelman Syndrome")

S4 "Intellectual Disabilit*" or "intellectual disorder*" or "Cri-du-Chat Syndrome*" or "down syndrome*" or "de lange syndrome*" or "Mental retardation*" or "Rubinstein?taybi syndrome*" or "trisomy 13 syndrome*" or "WAGR syndrome*" or "Williams Syndrome*" or "Prader?willi syndrome*" or "genetic disorder*" or "Fragile X*" or Rett* or Angelman* or "cat cry syndrome*" or "happy puppet syndrome*"

S5 (MH "Developmental Disabilities") OR "developmental disabilit*"

S6 "learning disabilit*"

S7 (MH "Intellectual Disability+")

S8 "mental handicap*" or "Mental deficien*" or "mental retard*"

S9 S1 OR S2 OR S3 OR S4 OR S5 OR S6 OR S7 OR S8

S10 (MH "Primary Health Care") OR "Primary* care*"

S11 (MH "Preventive Health Care+") OR "Preventive Medicine*"

S12 (MH "Family Practice") OR "General Practice*" or "family practice*" or "GP service*" or "family doctor*" or "family medicine*"

S13 (MH "Health Services for Persons with Disabilities")

S14 (MH "Health Services, Indigenous")

S15 "health service*" or "health services for persons with disabilit*" or "Indigenous health service*" or "preventive health service*"

S16 (MH "Community Health Services+") OR (MH "Community Mental Health Services+") OR "community* health service*"

S17 (MH "Health Services, Indigenous") OR "Aboriginal health service*" or "indigenous health service*"

S18 (MH "Nurse Practitioners+")

S19 (MH "Family Nurse Practitioners")

S20 "nurse practitioner*" or "family nurse practitioner*"

S21 "primary care nurs*"

S22 (MH "Physicians, Family") or "family physician*"

S23 S10 OR S11 OR S12 OR S13 OR S14 OR S15 OR S16 OR S17 OR S18 OR S19 OR S20 OR S21 OR S22

S24 "Health check*"

S25 "Health assessment*"

S26 "Preventive health check*"

S27 Health Screen*" OR AB "Health Screen*

S28 S24 OR S25 OR S26 OR S27

S29 S9 AND S23 AND S28

**Database 5: Scopus (Elsevier) Coverage 1996 – Present**

( TITLE-ABS-KEY ( "Intellectual Disabilit*" OR "Intellectual Disorder*" OR "Cri-Du-Chat Syndrome*" OR "Down Syndrome*" OR "De Lange Syndrome*" OR "Mental Retardation*" OR "Rubinstein?Taybi Syndrome*" OR "Trisomy 13 Syndrome*" OR "WAGR Syndrome*" OR "Williams Syndrome*" OR "Prader?Willi Syndrome*" OR "Genetic Disorder*" OR "Fragile X*" OR rett* OR angelman* OR "Developmental Disabilit*" OR "Learning Disabilit*" OR "Mental Handicap*" OR "Mental Deficien*" OR "Mental Retard*" OR "Cat Cry Syndrome*" OR "Happy Puppet Syndrome*" ) AND TITLE-ABS-KEY ( "Primary* Care*" OR "Preventive Medicine*" OR "General Practice*" OR "Family Practice*" OR "GP Service*" OR "Family Doctor*" OR "Family Medicine*" OR "Health Service*" OR "Health Services For Persons With Disabilit*" OR "Indigenous Health Service*" OR "Preventive Health Service*" OR "Aboriginal Health Service*" OR "Indigenous Health Service*" OR "Nurse Practitioner*" OR "Family Nurse Practitioner*" OR "Primary Care Nurs*" OR "Family Physician*" ) AND TITLE-ABS-KEY ( "Health Check*" OR "Health Assessment*" OR "Preventive Health Check*" OR "Health Screen*" ) )

**Database 6: Web Of Science (Clarivate) Coverage 1900 – Present**

1 "Intellectual Disabilit*" OR "Intellectual Disorder*" OR "Cri-Du-Chat Syndrome*" OR "Down Syndrome*" OR "De Lange Syndrome*" OR "Mental Retardation*" OR "Rubinstein?Taybi Syndrome*" OR "Trisomy 13 Syndrome*" OR "WAGR Syndrome*" OR "Williams Syndrome*" OR "Prader?Willi Syndrome*" OR "Genetic Disorder*" OR "Fragile X*" OR Rett* OR Angelman* OR "Developmental Disabilit*" OR "Learning Disabilit*" OR "Mental Handicap*" OR "Mental Deficien*" OR "Mental Retard*" OR "Cat Cry Syndrome*" OR "Happy Puppet Syndrome*" (Topic)

2 "Primary* Care*" OR "Preventive Medicine*" OR "General Practice*" OR "Family Practice*" OR "GP Service*" OR "Family Doctor*" OR "Family Medicine*" OR "Health Service*" OR "Health Services For Persons With Disabilit*" OR "Indigenous Health Service*" OR "Preventive Health Service*" OR "Aboriginal Health Service*" OR "Indigenous Health Service*" OR "Nurse Practitioner*" OR "Family Nurse Practitioner*" OR "Primary Care Nurs*" OR "Family Physician*" (Topic)

3 "Health Check*" OR "Health Assessment*" OR "Preventive Health Check*" OR “Health Screen*” (Topic)

4 #3 AND #2 AND #1
